# Hypolipidemic, anti‐inflammatory, and anti‐atherosclerotic effects of tea before and after microbial fermentation

**DOI:** 10.1002/fsn3.2096

**Published:** 2021-01-04

**Authors:** Xiujuan Deng, Yan Hou, Hongjie Zhou, Yali Li, Zhiqiang Xue, Xiaoting Xue, Ganghua Huang, Kunlun Huang, Xiaoyun He, Wentao Xu

**Affiliations:** ^1^ College of Food Science and Technology Yunnan Agricultural University Kunming China; ^2^ College of Long Run Pu‐erh Tea Yunnan Agricultural University Kunming China; ^3^ Key Laboratory of Precision Nutrition and Food Quality Department of Nutrition and Health China Agricultural University Beijing China

**Keywords:** anti‐atherosclerotic, anti‐inflammatory, hypolipidemic, microbial fermentation, tea

## Abstract

**Background:**

Microbial fermentation significantly affects the flavor and efficacy of tea. It is generally believed that fermented tea is more effective in lowering lipids, while unfermented tea can more effectively inhibit inflammation. However, there is not sufficient evidence to support this claim. To systematically compare the hypolipidemic, anti‐inflammatory, and anti‐atherosclerotic effects of tea before and after microbial fermentation, hyperlipidemic rats and inflammatory injury cells were treated with *Monascus purpureus*‐fermented pu‐erh tea water extract (MPT) and sun‐dried green tea water extract (SGT), respectively.

**Results:**

MPT, with higher levels of theabrownins, flavonoids, gallic acid (GA), and lovastatin, was more effective in reducing serum triglyceride (TG), total cholesterol (TC), low‐density lipoprotein cholesterol (LDL‐C), and inflammatory cytokines (TNF‐α, IL‐1β, and IL‐6), while SGT, with higher levels of tea polyphenols, amino acids, (‐)‐epigallocatechin gallate (EGCG), and theaflavins, was more effective in increasing serum high‐density lipoprotein cholesterol (HDL‐C) in hyperlipidemic rats. The foam cells on the arterial wall of the rats in the MPT group were visibly less, and the thrombosis time was longer than that in the SGT group. Cell experiments showed that MPT was more effective in protecting endothelial cells from damage than SGT.

**Conclusion:**

Surprisingly, *Monascus purpureus*‐fermented pu‐erh tea not only had better hypolipidemic and anti‐atherosclerotic effects than its raw material (sun‐dried green tea), but also was superior in anti‐inflammatory effects to the latter, which was possibly attributable to the great changes in functional ingredients during microbial fermentation.

## Graphical abstract

Surprisingly, Monascus purpureus‐fermented pu‐erh tea not only had better hypolipidemic and anti‐atherosclerotic effects than its raw material (sun‐dried green tea), but also was superior in anti‐inflammatory effects to the latter, which was attributable to the great changes in functional ingredients during microbial fermentation.

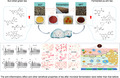

## 1 INTRODUCTION

With rapid societal development and changes in human lifestyle, nutritional metabolic syndrome represented by hyperlipidemia has become an urgent global problem. Inflammation response is an important immune defense mechanism of the body but can also induce various inflammatory diseases and even trigger "inflammation storm." Both hyperlipidemia and inflammation increase the risk of cardiovascular diseases, such as atherosclerosis. Cardiovascular disease is the leading cause of death in the world (Lu et al., 2019; Ueda et al., 2017). However, up to now, most drugs for hyperlipidemia, inflammation, and atherosclerosis have presented various limitations, such as undesirable side effects or high recurrence rates (Manning et al., 2014), prompting researchers to explore natural sources, such as tea, to address these diseases.

As a precious treasure of the Chinese nation, tea has been regarded as "the patron saint of life" for its health benefits, such as its detoxifying, anti‐aging, and life extension properties. With desirable flavor, high nutritional value, and unique health benefits, tea has become one of the world's most widely consumed beverages. Pu‐erh tea is popular with consumers and is made by further processing sun‐dried green tea from the fresh leaves of *Camellia sinensis var Assamica,* which is widely produced in the Yunnan Province of China. Pu‐erh tea is categorized into unfermented and fermented pu‐erh tea, depending on whether it undergoes a microbial fermentation process, which significantly changed pu‐erh tea quality. After microbial fermentation, the tea's aroma changes from fragrant to a unique aged aroma, while the liquor color changes from yellow‐green to reddish‐brown, and the taste changes from strong to mellow and smooth. Tea polyphenols are transformed into new active ingredients, such as theabrownin via a series of complicated chemical reactions, including oxidation, decomposition, and polymerization (Zhu et al., 2020), which significantly impacts the efficacy of pu‐erh tea.

In recent years, studies have revealed that pu‐erh tea displays extensive hypolipidemic, antihypertensive, hypoglycemic, antimicrobial, and anti‐atherosclerotic bioactivity (Cai et al., 2017; Chu et al., 2011; Q. Sun & Yan, 2014). It is generally believed that fermented pu‐erh tea can prevent obesity and reduce blood lipids more effectively, while sun‐dried green tea and unfermented pu‐erh tea have a better anti‐inflammatory effect. However, there is not sufficient evidence to support this claim. Hou et al., (2009), Jiang and Shao (2011), and Xiong, Peng et al. (2019) studied the effects of fermented and unfermented pu‐erh tea on the lipid levels in hyperlipidemic rats, but their results were not consistent. Previous studies regarding anti‐inflammatory tea activity mainly focused on green tea and black tea, or the major components, such as the (‐)‐epigallocatechin gallate (EGCG) of green tea. Cell experiments (Li et al., 2011; Zhang et al., 2012) showed that both unfermented and fermented pu‐erh tea exhibit a certain degree of anti‐inflammatory activity. Chen et al., (2016) and Xiao et al. (2019) found that pu‐erh tea significantly reduced the atherosclerosis of ApoE−/− mice. No comparative studies have been performed regarding the health efficacy of tea before and after microbial fermentation.

**FIGURE 1:**
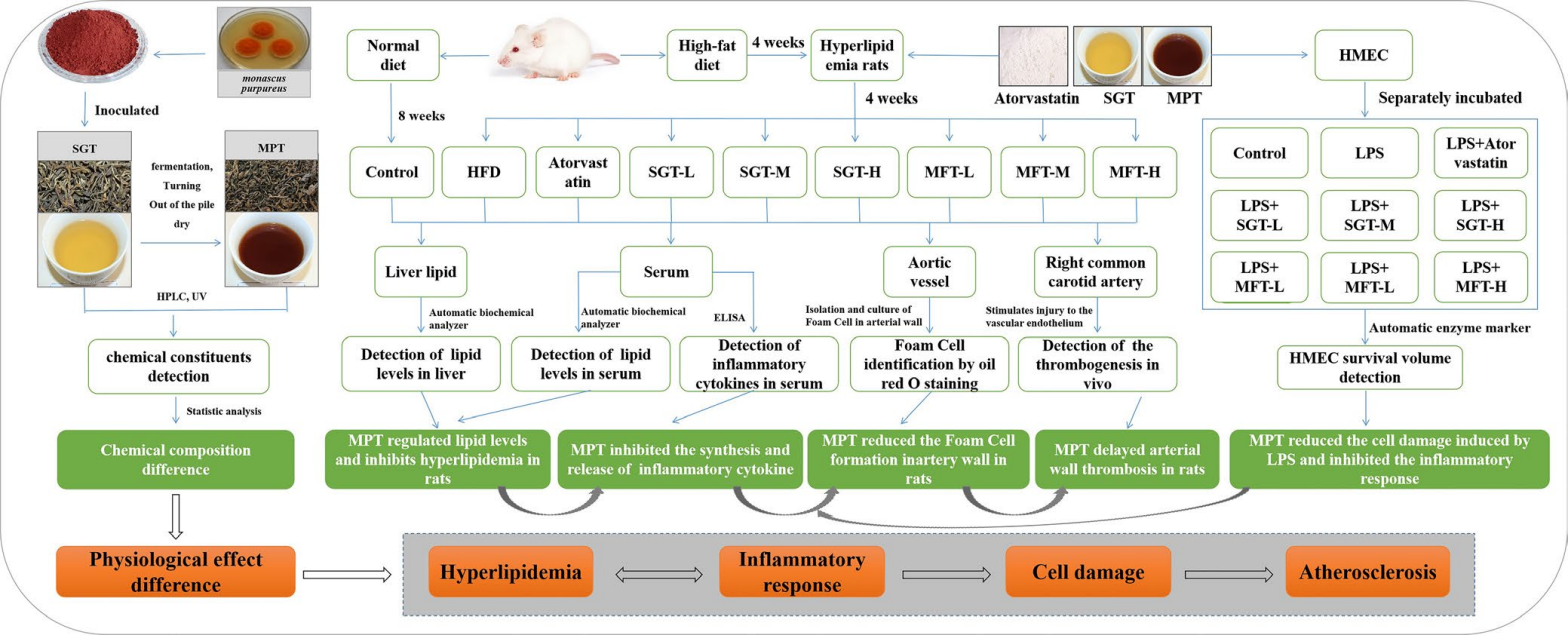
Technical route. SGT: sun‐dried green tea water extract, MPT: *Monascus purpureus*‐fermented pu‐erh tea water extract, HMEC: human micro‐artery endothelial cell, HFD: high‐fat diet, SGT‐L: low‐dose SGT, SGT‐M: medium‐dose SGT, SGT‐H: high‐dose SGT, MPT‐L: low‐dose MPT, MPT‐M: medium‐dose MPT, MPT‐H: high‐dose MPT, LPS: lipopolysaccharide

Therefore, to systematically compare the health benefits of tea before and after microbial fermentation in this study, the sun‐dried green tea is inoculated with *Monascus purpureus*, a fungus strain capable of generating high levels of lovastatin (Li et al., 2013), to obtain fermented pu‐erh tea. A hyperlipidemic rat model and an inflammatory injury cell model are constructed to investigate the regulatory effect of their water extracts on hyperlipidemia, inflammation, and atherosclerosis. The overall technical route is shown in Figure 1.

## 2 MATERIALS AND METHODS

### 2.1 Preparation of MPT and SGT

The sun‐dried green tea, provided by the Kunming Wanhongyang Tea Co. Ltd. (Yunnan, China), was inoculated with *Monascus purpureus* MPT13 (independent patent, 201010182965.9) and fermented to obtain the *Monascus purpureus*‐fermented pu‐erh tea. The tea samples were soaked in boiling water and filtered (1:50, 10 min, five times), after which the filtrate was vacuum‐concentrated, freeze‐dried, and refrigerated at 4°C for later use.

Composition analysis. The tea polyphenol content was measured using the Folin‐phenol method, the total free amino acids were measured using the ninhydrin method, while the anthrone–sulfuric acid colorimetric assay was used to measure the soluble sugar content, and the flavonoid content was determined using the aluminum trichloride colorimetric method. The theaflavin, thearubigin, and theabrownins contents were measured according to a method described in a previous report (Wang et al., 2011). The caffeine, GA, and catechin levels were determined using high‐performance liquid chromatography (HPLC) (Zhao et al., 2014). The lovastatin levels were measured according to a method described in a previous report (Li et al., 2013).

### 2.2 Animal experiments

#### 2.2.1 Establishment of the hyperlipidemia rat model

Healthy SPF inbred Sprague Dawley rats (eight weeks old, 180.0–236.0 g) were purchased from the animal laboratory center of the Peking University Health Science Center (animal license number: scxk‐2009). The rats were housed in a controlled environment (22 ± 2°C, 40%–60% humidity, and a 12‐hr light–dark cycle), 4 rats per cage, and provided free access to water and fodder. They were acclimatized for 7 d before commencing with the experiment. Then, 12 rats were randomly selected as the control group and intragastrically administrated distilled water, while the remaining rats (HFD group) received daily doses of intragastrically administrated natural animal fat at 1.0 g/kg.bw. After four weeks, 0.5 ml blood was collected from the tail veins of all the rats, and the serum triglyceride and TC contents were determined using an AU2700 automatic biochemical analyzer according to the instructions of the commercial assay kits (Beckman Coulter). The triglyceride and TC levels in the HFD group were significantly higher than in the control group.

#### 2.2.2 Grouping and treatment methods

The hyperlipidemic rats were randomly divided into eight groups (12 rats per group). Atorvastatin calcium tablets (Lipitor) were used as a positive control drug and were purchased from Pfizer (USA). Since the start of the fifth week, the rats in the atorvastatin group received daily intragastric doses of atorvastatin at 4.13 mg/kg.bw daily. The rats in the MPT‐L, MPT‐M, and MPT‐H groups, as well as the SGT‐L, SGT‐M, and SGT‐H groups, were intragastrically administered different doses of MPT or SGT. The low, medium, and high doses were 125 mg/kg.bw, 250 mg/kg.bw, and 500 mg/kg.bw, respectively, which were converted to the intragastric doses for the rats according to the recommended amount for humans. The rats in the HFD and the control groups were continuously given an equal volume of distilled water via gavage for four weeks.

#### 2.2.3 Biochemical analyses of the serum and liver parameters

All the rats were anesthetized and sacrificed. The orbital blood of the rats was collected and centrifuged at 1157.13 *g* for 10 min. The serum was transferred to an Eppendorf tube and stored at −20°C for further analysis. A chloroform/methanol solution (1/1, V/V) was used to extract the lipids from the liver tissue, after which the mixed solution was centrifuged at 1,000 × g for 10 min. The supernatant was transferred to an Eppendorf test tube and stored at −20°C for further analysis.

The TC, TG, HDL‐C, and LDL‐C levels were measured using an AU2700 automatic biochemical analyzer according to the instructions of the commercial assay kits (Beckman Coulter). The IL‐1β, IL‐6, and TNF‐α levels were determined using an ELISA kit (Shanghai Westang ExCell Biology Inc) according to the manufacturer's instructions.

#### 2.2.4 Determination of thrombosis in vivo

After four weeks of gavage treatment, all the rats were anesthetized and fixed on an aseptic operating table. The fur was removed from the neck of each rat, and the sites were disinfected. A central incision was made in the necks to separate the rats' right common carotid artery. An in vivo Thrombosis Test Kit and a BT87‐3 experimental intracorporeal thrombosis surveyor (Beijing, China) were used to measure the thrombosis time.

#### 2.2.5 Foam cell isolation and histological analysis

After completing the thrombosis test, the aortic vessels were rapidly separated, washed clean of residual blood using sterile Dulbecco's phosphate‐buffered saline (DPBS), and placed in a cell culture medium at 4°C and then in a cryo tube. Under an anatomical microscope, arterial vessels were cut open along the longitudinal axis, the vascular endothelial cells were scraped off gently, and subcutaneous tissue cells were retained. The isolated endovascular tissue cells were placed in RPMI‐1640 culture medium containing collagenase I after they were washed twice and incubated in a 5% CO_2_ incubator at 37°C for 30 min. After tissue cell digestion, the suspension was filtered and incubated in a 24‐well cell culture plate at 37°C in a 5% CO_2_ incubator for 120 min, after which the supernatant was discarded. The foam cells were identified using anti‐rat CD11b Alexa Fluor® 488 (eBioscience Inc) and detected with an UltraVIEW VoX high‐speed laser confocal imaging system (PerkinElmer Inc.). The anti‐rat CD11b Alexa Fluor® 488‐positive cells were selected for Oil Red O (Sigma‐Aldrich Inc) staining.

### 2.3 Cell experiments

The HMECs were purchased from the ATCC center in the United States. The cells were inoculated into 96‐well plates and cultured in an incubator with 5% CO_2_ at 37°C for 24 hr. The cells were randomly divided into nine groups. The medium for the endothelial cells consisted of 199/EBSS (Hyclone Inc), 10% fetal bovine serum (FBS, Gibco Inc), streptomycin (100 μg/ml), and penicillin (100 IU/ml). The HMECs were cultured in 96‐well plates containing culture medium to which LPS was added, except for the control group. All the HMECs were incubated in an incubator with 5% CO_2_ at 37°C for 2 hr, after which MPT, SGD, and atorvastatin were added. The final concentrations of LPS and atorvastatin were 1 μg/ml and 4.13 μg/ml, respectively. The MPT and SGT were 1.25 μg/ml, 2.50 μg/ml, and 5.00 μg/ml, respectively.

The HMECs of each group were incubated in an incubator with 5% CO_2_ at 37°C for 24 hr, after which MTT (Sigma Breakthrough Technologies Inc.) solution was added at a final concentration of 1.0 g/ml. All the cells were washed twice with serum‐free medium and incubated in an incubator with 5% CO_2_ at 37°C for 2 hr. All the supernatant was removed, and the cells were washed twice with cell culture medium at 100 μl/well. Then, 100 µl dimethyl sulfoxide (DMSO, Sigma‐Aldrich Inc.) was added to each well, and the plate was subjected to detection at 570 nm using a THERMO Multiskan MK3 automatic microplate reader (Thermo Scientific Inc.).

### 2.4 Statistical analysis

All data are expressed as the mean ± *SD*. Differences between groups were evaluated using one‐way ANOVA and Tukey's post hoc test or independent‐samples *t* test (SPSS Statistics 25.0). Each experiment was repeated at least three times.

## 3 RESULTS

### 3.1 The main functional ingredients of SGT and MPT

The main functional ingredients of SGT and MPT were identified to investigate the differences in tea's physiological activity before and after fermentation. Compared with SGT, as shown in Table 1, the tea polyphenols, free amino acids, soluble sugar, theaflavins, thearubigins, catechins, EGC, and EC content in MPT were significantly reduced, while the flavonoids, theabrownins, and GA content were significantly higher. Furthermore, EGCG and ECG were not detected in MPT, while lovastatin was not present in SGT. No significant changes were found in the caffeine content in MPT compared with SGT. These results revealed that tea's functional ingredients changed substantially before and after microbial fermentation.

**TABLE 1:** The main functional ingredients of MPT and SGT

Constituents	SGT (%)	MPT (%)	Amplitude of variation
Free amino acids	2.71 ± 0.24	1.06 ± 0.02*	−60.89%
Flavonoids	2.21 ± 0.25	4.09 ± 0.05*	89.52%
Soluble sugar	12.96 ± 0.96	6.78 ± 0.62*	−47.69%
Theaflavins	4.97 ± 0.07	2.15 ± 0.10*	−56.74%
Thearubigins	3.87 ± 0.11	1.47 ± 0.21*	−62.02%
Theabrownins	1.71 ± 0.24	8.65 ± 0.11*	405.85%
Tea polyphenols	27.29 ± 1.49	11.47 ± 0.22*	−57.97%
Catechins	24.21 ± 0.46	5.16 ± 0.30*	−78.69%
(‐)‐epicatechin (EC)	1.38 ± 0.30	0.32 ± 0.50*	76.81%
(‐)‐epigallocatechin (EGC)	1.46 ± 0.10	0.43 ± 0.40*	−70.55%
(‐)‐epicatechin gallate (ECG)	3.64 ± 0.11	ND	‐
(‐)‐epigallocatechin gallate (EGCG)	4.22 ± 0.24	ND	‐
Gallic acid (GA)	0.12 ± 0.03	0.38 ± 0.08*	216.67%
Caffeine	3.70 ± 0.23	3.62 ± 0.20	−2.16%
Lovastatin	ND	0.011 ± 0.006	‐

Note

All data are presented as mean ± *SD*.

* *p* < .05. ND means no detection.

### 3.2 MPT was more effective than SGT in inhibiting hyperlipidemia

**FIGURE 2:**
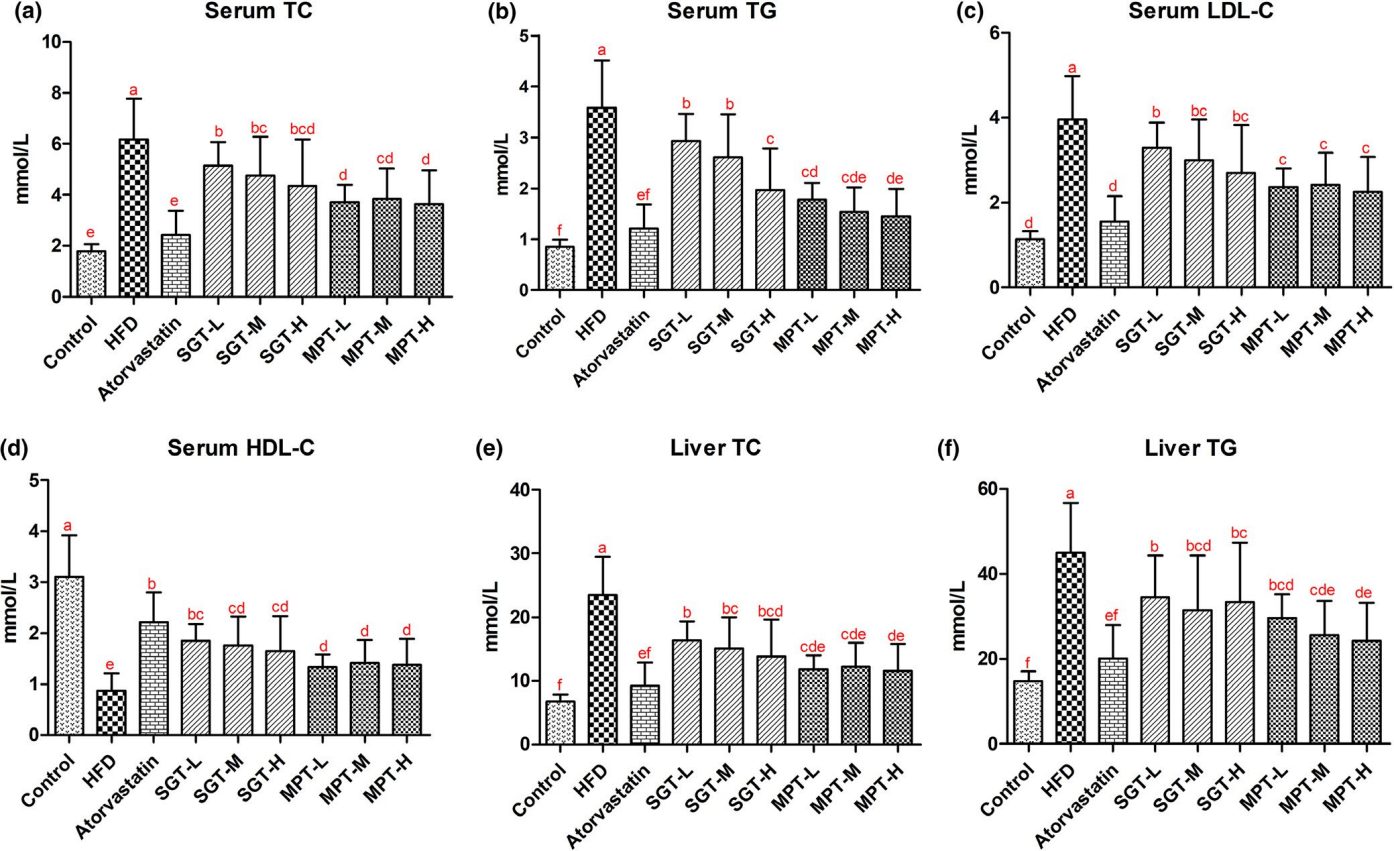
MPT and SGT inhibited hyperlipidemia. The levels of (a) serum TC, (b) serum TG, (c) serum LDL‐C, (d) serum HDL‐C, (e) liver TC, and (f) liver TG in hyperlipidemic rats. All data are presented as the mean ± *SD* (*n* = 12). The different lowercase letters indicate significant differences (*p* < .05)

An HFD can easily cause hyperlipidemia and other chronic metabolic diseases. After the rats were fed an HFD, the serum TC, TG, and LDL‐C concentrations in the HFD group were significantly higher than those in the control group (*p* < .001), while the concentration of HDL‐C was substantially lower (*p* < .001) (Figure 2). Both MPT and SGT significantly reduced the serum TC, TG, and LDL‐C of the animals (Figure 2a‐c). The serum TC, TG, and LDL‐C concentrations in the MPT group decreased by 37.75%–41.17%, 50.25%–59.43%, and 38.73%–43.01% (*p* < .001), respectively, while those in the SGT group decreased by 16.62%–29.47%, 18.08%–45.29%, and 16.64%–31.69%, respectively (*p* < .050) (Table S1‐S3). The inhibitory effect of MPT on TC, TG, and LDL‐C was superior to SGT, and the differences were statistically significant at low doses (*p* < .01).

On the contrary, MPT and SGT significantly improved the levels of serum HDL‐C in hyperlipidemic rats. The serum HDL‐C concentration in the MPT group increased by 52.91%–62.73%(*p* < .05), while it was 89.37%–112.07% higher in the SGT group (*p* < .001) (Figure 2d, Table S4). The promotional effect of SGT on HDL‐C surpassed that of MPT, and the differences were statistically significant at low doses (*p* < .05). Furthermore, the HDL‐C level in the SGT‐L group was close to that of the atorvastatin group (*p* > .05).

The liver is the primary site for lipogenesis and lipid metabolism. The TC and TG concentrations in the livers of the hyperlipidemic rats in the HFD group were significantly higher than the normal rats in the control group (*p* < .001) (Figure 2e‐f). The liver TC and TG levels in the hyperlipidemic rats fed with MPT and SGT were significantly reduced compared with those in the HFD group (*p* < .001). The liver TC and TG concentrations in the MPT group decreased by 47.91%–50.77% and 34.06%–46.06%, respectively, while those in the SGT group decreased by 30.23%–40.98% and 23.41%–30.03%, respectively (Table S5–6 in Supplementary Material). The inhibitory effect of MPT on liver TC and TG was better than that of SGT, and the difference was statistically significant at low and high doses (*p* < .050). Furthermore, the liver lipid levels in the MPT group were close to the atorvastatin group (*p* > .050). Therefore, MPT exhibited a superior hypolipidemic effect to SGT in rats.

### 3.3 MPT performed better in inhibiting inflammatory damage than SGT and atorvastatin

**FIGURE 3:**
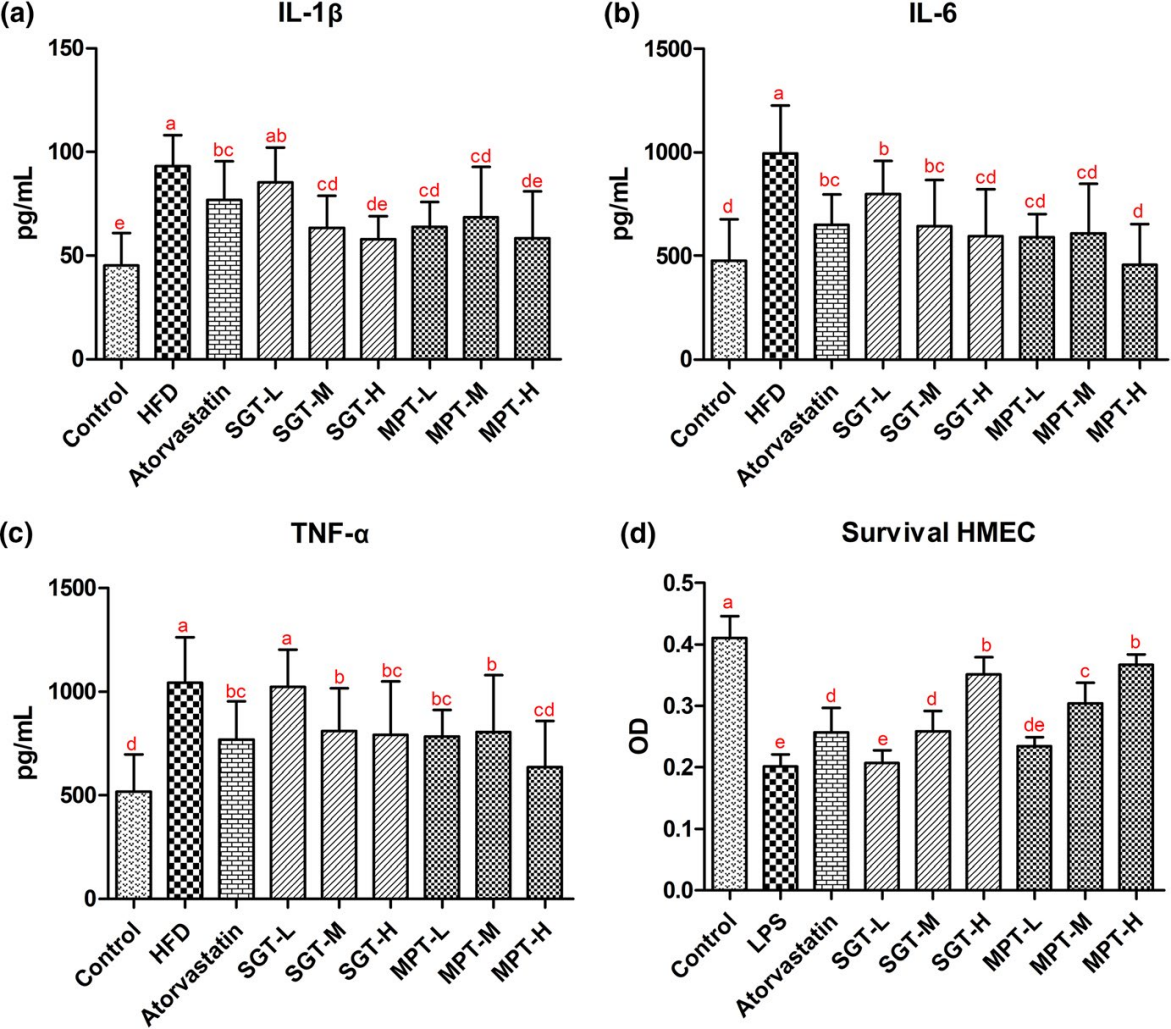
MPT and SGT attenuated the inflammatory response and protected endothelial cells from damage. The levels of serum inflammatory cytokines (a) IL‐1β, (b) IL‐6, (c) TNF‐α, and (d) the survival HMEC. All data are presented as the mean ± *SD*. The different lowercase letters indicate significant differences (*p* < .05)

The occurrence of hyperlipidemia is usually accompanied by an increase in the levels of inflammatory cytokines. Therefore, the changes in inflammatory cytokine levels in the serum of hyperlipidemic rats were further examined to evaluate the effect of MPT on inflammation. The levels of the major inflammatory cytokines (IL‐1β, IL‐6, and TNF‐α) in the HFD group were significantly higher than those in the control group (*p* < .001) (Figure 3a‐c), indicating that an HFD stimulated their release.

After MPT and SGT treatment, the serum IL‐1β, IL‐6, and TNF‐α levels in the hyperlipidemic rats were reduced. The IL‐1β, IL‐6, and TNF‐α concentrations in the MPT group decreased by 26.29%–37.31% (*p* < .001), 38.91%–54.04% (*p* < .001), and 22.77%–39.15% (*p* < .010), respectively, while those in SGT group decreased by 8.29%–37.67% (*p* > .050, *p* < .001), 19.75%–35.47% (*p* < .050), and 2.02%–24.12% (*p* > .050, *p* < .010), respectively (Table S7–S9). The anti‐inflammatory response in the MPT group regarding the IL‐1β, IL‐6, and TNF‐α levels was better than in the SGT group, while the differences were statistically significant at low doses. Furthermore, the IL‐1β level in the MPT‐H and SGT‐H groups was significantly lower than that in the atorvastatin group (*p* < .010), while it was close to that in the control group (*p* > .050). The IL‐6 level in the MPT‐H group was lower than in the atorvastatin group (*p* < .050) and the control group (*p* > .050). The TNF‐α level in the MPT‐H group was close to that in the control group (*p* > .050). These results indicated that MPT and SGT both inhibited the release of inflammatory cytokines in the serum of hyperlipidemic rats and that MPT performed better than SGT and atorvastatin.

To further explore the effect of MPT and SGT on vascular endothelial cell damage caused by the inflammatory response, LPS was used to stimulate this response in HMEC, with the intervention of atorvastatin, MPT, and SGT, respectively. As shown in Figure 3d and Table S10, LPS induced severe damage and apoptosis in HMEC, and the survival rate of these cells in the LPS group was significantly lower than in the control group (*p* < .001). However, MPT and SGT both mitigated this damage. The survival level of HMEC in the MPT and SGT groups was 16.66%–82.59% and 2.75%–74.30% higher than in the LPS group in a dose‐dependent manner. Therefore, the protective effect of MPT on HMEC was better than SGT and atorvastatin, and the differences were statistically significant at medium doses (*p* < .010).

### 3.4 MPT was more effective than SGT in reducing atherosclerotic lesions

Thrombosis is one of the most critical manifestations of atherosclerosis. The formation of foam cells, the characteristic pathological cells in atherosclerotic plaques, represents the first thrombosis and atherosclerosis event. Therefore, to explore the influence of hyperlipidemia on atherosclerosis and MPT's intervention efficacy, atherosclerotic lesion experiments were performed in isolated rats. Using in vivo thrombogenic reagent and weak electric current stimulation, the blood vessel substances were decomposed into strong oxygen free radicals, which specifically damaged the vascular endothelial cells, inducing additional foam cells and initiating thrombosis.

**FIGURE 4:**
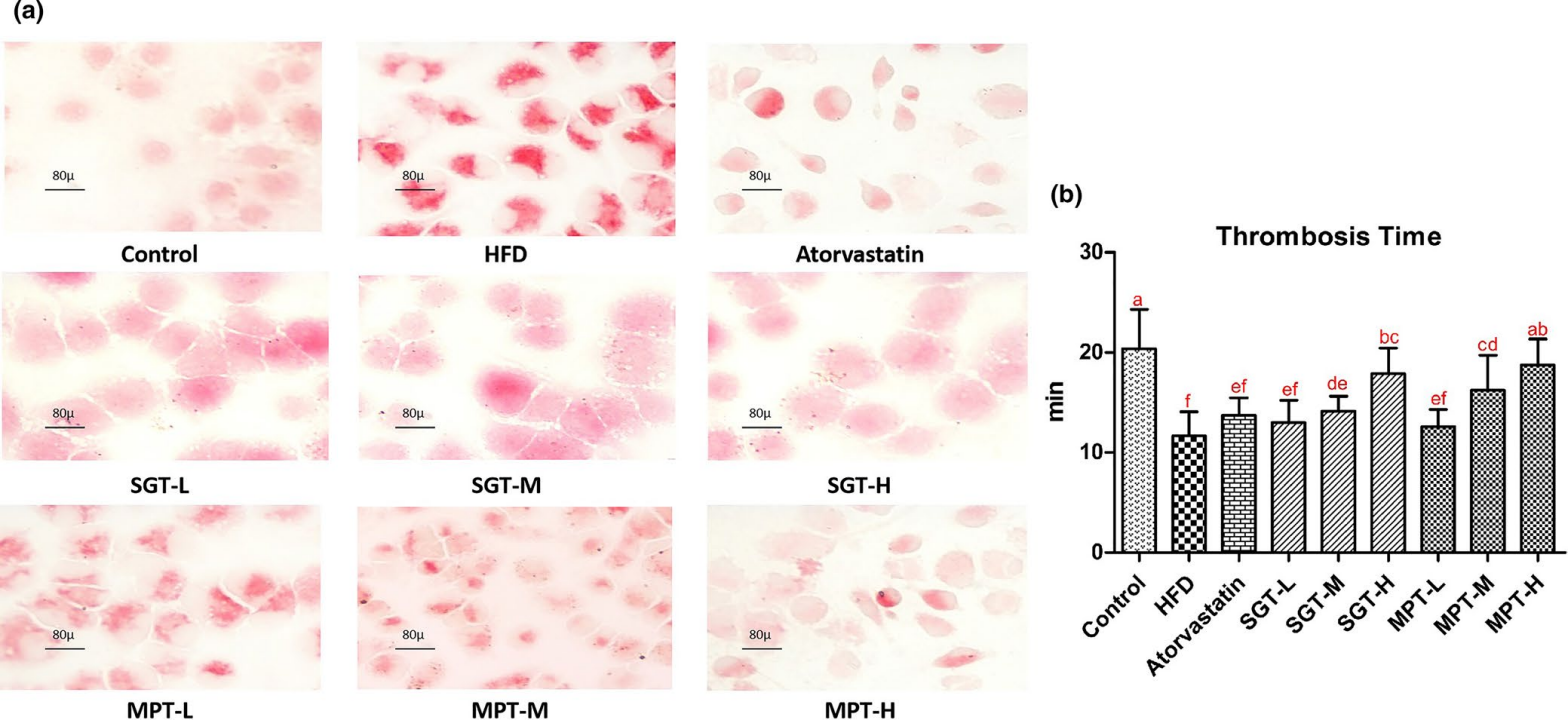
MPT reduced foam cells and inhibited thrombosis to anti‐atherosclerosis. (a) ORO staining of the foam cells from the rats under different treatments. The magnification was × 40. (b) The thrombosis time of the rats under different treatments. All data are presented as the mean ± *SD* (*n* = 12). The different lowercase letters indicate significant differences (*p* < .05)

The results showed that the foam cell area under the arterial intima of the rats in the HFD group was significantly increased (*p* < .05) compared with that in the control group (Figure 4a), indicating that hyperlipidemia aggravated the formation of foam cells in the arterial walls of the rats. However, MPT, SGT, and atorvastatin alleviated this condition since the foam cell areas in these groups were significantly smaller than in the HFD group (*p* < .050). Furthermore, the inhibitory effect of MPT on the foam cells was visibly superior to the performance of SGT, and even better than that in the control group. Figure 4b shows that the thrombosis time of the HFD group was 8.690 min less than in the control group (*p* < .001), indicating that hyperlipidemia facilitated thrombosis to a certain extent. However, MPT and SGT alleviated this situation. Compared with the HFD group, the thrombosis time of the hyperlipidemic rats in the MPT‐L, MPT‐M, and MPT‐H groups was prolonged by 7.63% (*p* > .050), 39.13% (*p* < .001), and 60.66% (*p* < .001), respectively, while that in the SGT‐L, SGT‐M, and SGT‐H groups was prolonged by 11.45% (*p* > .050), 21.16% (*p* < .050), and 52.97% (*p* < .001), respectively (Table S11).

Overall, hyperlipidemia exacerbated atherosclerotic lesions and facilitated the formation of foam cells and thrombosis in the artery wall, while MPT exhibited outstanding inhibitory and enhancing impact in a dose‐dependent manner, which was more efficacious compared with SGT and atorvastatin.

## 4 DISCUSSION

The long‐term intake of an HFD can lead to an imbalance between energy intake and energy expenditure, which eventually leads to hyperlipidemia, inducing vascular endothelial injury and lipid deposition, thereby increasing the risk of atherosclerosis. Pu‐erh tea effectively lowers lipids, as evidenced by epidemiology, in vitro experiments, and clinical investigation (Huang et al., 2019; Sun et al., 2019). Furthermore, theabrownins, tea polysaccharides, tea polyphenols, GA, caffeine, and lovastatin can lower lipids (Gong et al., 2010; Nakamura et al., 2017; Sugiura et al., 2020; Xiong, Cao et al., 2019; Zeng et al., 2019; Zhong et al., 2020). In this study, sun‐dried green tea was inoculated with *Monascus purpureus* and fermented to obtain fermented pu‐erh tea. It was confirmed that both these water extracts inhibited hyperlipidemia, but their mechanisms, as well as their primary functional and active ingredients, were different. MPT exhibited a higher level of theabrownins, tea flavonoids, GA, and lovastatin, while it was more effective in reducing liver and serum TC, TG, and LDL‐C. SGT displayed higher levels of tea polyphenols, catechins, EGCG, theaflavins, and thearubigins and was more effective in increasing serum HDL‐C in hyperlipidemic rats. Overall, MPT displayed a better hypolipidemic effect than its raw material without fermentation.

Green tea, yellow tea, white tea, oolong tea, and unfermented pu‐erh tea all display anti‐inflammatory properties, which is generally considered the contribution of tea polyphenols (especially EGCG), theanine, and theaflavins (Li et al., 2017; De Lima Mota et al., 2015). Tea polyphenols can interfere with NF‐B, MAPKs, and the notch signal transduction pathway, inhibiting the secretion and gene expression of cytokines, such as TNF‐α, IL, NO, and PGE2 (Wang et al., 2017). Theanine can inhibit the secretion of TNF‐α, IL‐1β, IL‐6, and iNOS inflammatory cytokines and down‐regulate the inflammation level (Min et al., 2020). Theaflavins can effectively restrict the activity of LPS‐induced TNF‐α, IL‐1β, and IL‐6, as well as the expression of JNK and p38 MAPK mitogen‐activated protein kinase, thus exerting an anti‐inflammatory effect (Wu et al., 2017). However, little comparative studies are available regarding the anti‐inflammatory impact of teas made from the same raw materials but processed differently.

The results of this study clearly showed that MPT could attenuate inflammatory response and protect endothelial cells from damage, evidenced by reduced TNF‐α, IL‐1β, and IL‐6 and increased HMEC, surpassing the performance of SGT and atorvastatin. Contrary to SGT, EGCG, which is believed to play a substantial anti‐inflammatory role in tea, was not detected in MPT, and the content levels of other anti‐inflammatory ingredients, such as tea polyphenols, catechins, and theaflavin, were also significantly lower. These findings suggest that the anti‐inflammatory mechanism of tea is significantly different after microbial fermentation. The lovastatin, theabrownin, GA, and flavonoid content were significantly higher in MPT than in SGT. Studies indicated that caffeine, flavonoids, GA, and lovastatin also exhibited certain anti‐inflammatory effect qualities (Badshah et al., 2019; Choi et al., 2018; Indra et al., 2013; Liu et al., 2020; Tang et al., 2016) and that there is a close relationship between the anti‐inflammatory effect and the antioxidant pathway of tea. Theabrownins display strong antioxidant activity, which may play a role in the suppression of inflammation. Correlation analysis of the chemical composition and efficacy in this study showed that theabrownins, GA, and flavonoids exhibited a significant negative correlation with IL‐6, while GA and flavonoids were negatively correlated with TNF‐α (Table S12). Therefore, it is speculated that the higher beneficial effect of MPT can be mainly attributed to flavonoids, GA, and other characteristic active metabolites produced during tea fermentation.

The cause of atherosclerosis is complicated and involves endothelial damage, oxygen free radical damage, lipid composure, blood platelet aggregation, inflammatory cell infiltration, vascular smooth muscle cell calcium overload, pathological angiogenesis, partial thrombosis, and more. Although tea polyphenols, tea polysaccharides, dihydromyricetin, and caffeine are closely related to their anti‐atherosclerotic effect (Liu et al., 2017; Yang et al., 2020; Zhang et al., 2018, 2019), the specific action mechanism remains unclear.

In this study, high levels of inflammatory cytokines accelerated atherosclerosis in hyperlipidemic rats, while the intervention of MPT and SGT significantly reversed this condition. MPT was shown to be more prominent than SGT. Hyperlipidemia can cause lipid infiltration of the endothelium and cell damage, stimulating the release of inflammatory cytokines, promoting the occurrence of an inflammatory response, and accelerating the apoptosis of arterial endothelial cells, eventually leading to thrombosis. However, the inflammation in the liver, adipose tissue, and arterial blood wall increases the accumulation of lipids in the body, exacerbating hyperlipidemia. The abnormal changes in plasma lipids and lipoproteins can also affect platelets' functionality, changing the activity of the coagulation system, fibrinogen, and fibrinolysis system, promoting thrombosis, and accelerating the formation and development of atherosclerosis.

**FIGURE 5:**
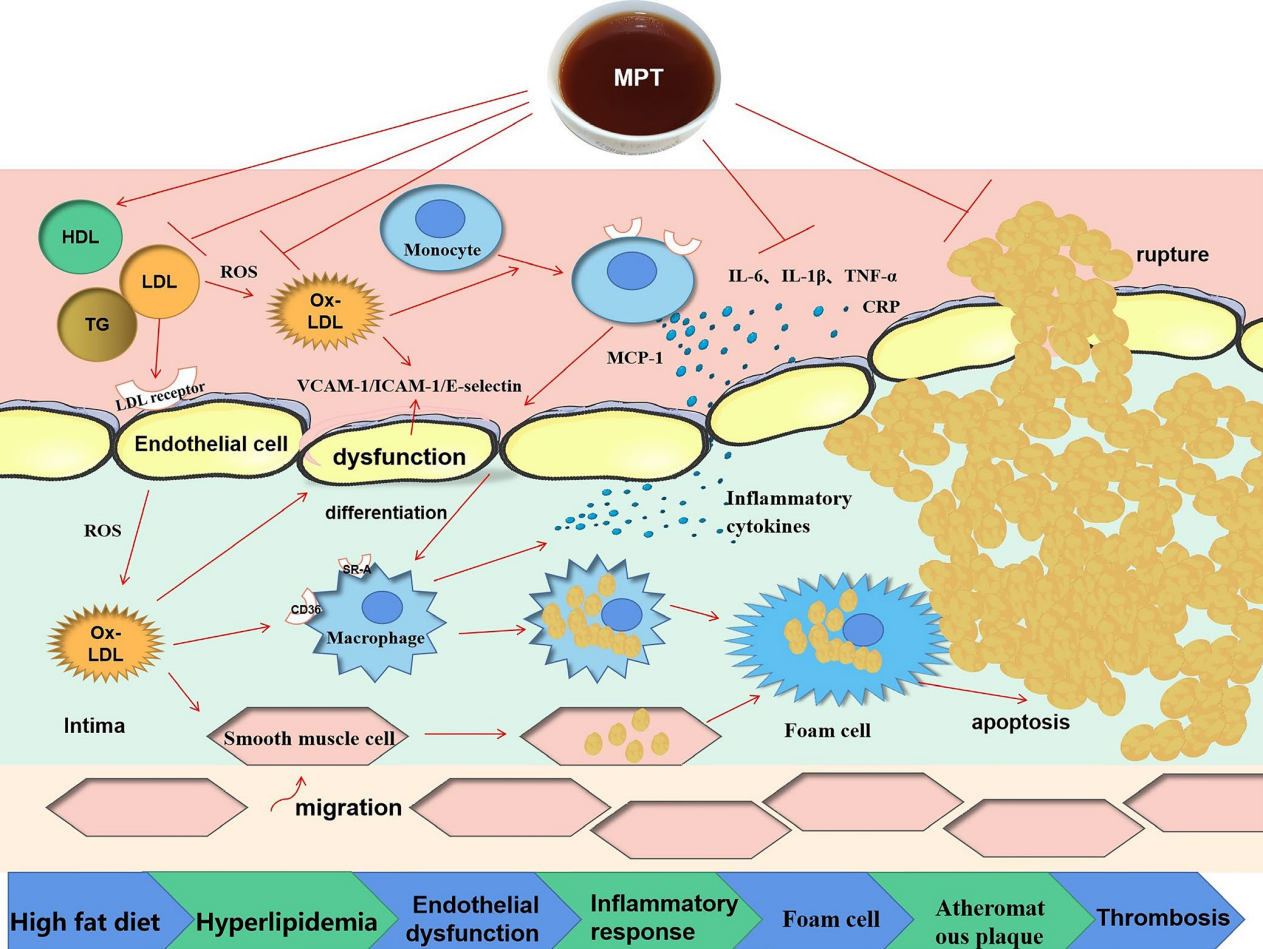
The proposed pathway involved in hypolipidemic, anti‐inflammatory, and anti‐atherosclerotic effects of MPT. The arrows indicate activation or induction, while the vertical lines indicate inhibition or obstruction

Therefore, it is speculated that the prominent anti‐atherosclerotic effect of MPT is also related to its hypolipidemic and anti‐inflammatory characteristics. The combined action of functional ingredients produced during the microbial fermentation process allows MPT to inhibit the inflammatory injury and cell apoptosis caused by high lipid infiltration and inflammatory response more effectively, preventing and inhibiting atherosclerosis. The proposed mechanism is shown in Figure 5. *Monascus purpureus* is a beneficial filamentous fungus containing many metabolites, such as lovastatin, *Monascus purpureus* polysaccharides, polyunsaturated fatty acids, γ‐aminobutyric acid, ergosterol, *Monascus purpureus* pigments, and flavonoids, which can reduce blood lipids, blood glucose, and blood pressure while exhibiting anti‐inflammatory, anti‐atherosclerotic, anticancer, and immunity‐enhancing properties (Cheng et al., 2011; Xiong, Cao, et al., 2019). Based on the remarkable anti‐inflammatory, anti‐atherosclerotic, and hypolipidemic effect of MPT, it is essential to identify the specific beneficial ingredients and regulatory mechanisms for developing new functional fermented tea products.

## 5 CONCLUSIONS

This study represents the first systematic comparative study on the anti‐inflammatory impact and other beneficial tea properties before and after microbial fermentation. After sun‐dried green tea was inoculated with *Monascus purpureus* for microbial fermentation, the functional ingredients display significant modification, leading to differences in the health advantages of SGT and MPT. MPT, with higher levels of theabrownins, flavonoids, gallic acid, and lovastatin, was more effective in inhibiting hyperlipidemia, inflammation, and atherosclerosis than SGT. Different from SGT, EGCG and other ingredients, which were believed to play a major anti‐inflammatory role in tea, were not detected or significantly lower in MPT. However, the anti‐inflammatory effect of MPT was better than SGT, indicating that the anti‐inflammatory mechanism of fermented pu‐erh tea differs from that of sun‐dried green tea. The combined action of functional ingredients produced during the microbial fermentation process enables MPT to effectively inhibit the vascular endothelial cell damage caused by hyperlipidemia and inflammatory response, preventing atherosclerosis. This study provides a solid scientific basis to utilize the beneficial microorganisms present during the fermentation process to develop new functional fermented tea products.

## CONFLICT OF INTEREST

The authors have declared no conflict of interest.

## 6 ETHICS STATEMENT

All the animal experimental procedures adhered to the Guidelines for the Care and Use of Laboratory Animals (Ministry of Science and Technology of the People's Republic of China) and were approved by the Ethical Committee for Animal Experiments, Yunnan Agricultural University.

## Supporting information

Supplementary Material

## Data Availability

The data that support the findings of this study are available in the supplementary material of this article, as well as available from the corresponding author upon reasonable request.
