# Supplementary material for: Hypolipidemic, anti‐inflammatory, and anti‐atherosclerotic effects of tea before and after microbial fermentation
Source: Food Sci Nutr. 2021 Jan 4;9(2):1160–70. doi: 10.1002/fsn3.2096 (PMC7866600; doi:10.1002/fsn3.2096)
Supplement: Supplementary file 1 — Supplementary Material [file FSN3-9-1160-s001.doc]

**Supplementary Material**

Table S1 One-way ANOVA of serum TC in rats.

|  | | Control | HFD | Atorvastatin | SGT-L | SGT-M | SGT-H | MPT-L | MPT-M | MPT-H |
| --- | --- | --- | --- | --- | --- | --- | --- | --- | --- | --- |
| Control | Mean difference | - | -4.388^*^ | -0.644 | -3.362^*^ | -2.966^*^ | -2.569^*^ | -1.927^*^ | -2.059^*^ | -1.848^*^ |
|  | Sig. | - | 0.000 | 0.205 | 0.000 | 0.000 | 0.000 | 0.000 | 0.000 | 0.000 |
| HFD | Mean difference | 4.388^*^ | - | 3.743^*^ | 1.026^*^ | 1.421^*^ | 1.818^*^ | 2.460^*^ | 2.329^*^ | 2.540^*^ |
|  | Sig. | 0.000 | - | 0.000 | 0.045 | 0.006 | 0.001 | 0.000 | 0.000 | 0.000 |
|  | Decreasing amplitude | -71.13% | - | -60.68% | -16.62% | -23.04% | -29.47% | -39.88% | -37.75% | -41.17% |
| Atorvastatin | Mean difference | 0.644 | -3.743^*^ | - | -2.717^*^ | -2.322^*^ | -1.925^*^ | -1.283^*^ | -1.414^*^ | -1.203^*^ |
|  | Sig. | 0.205 | 0.000 | - | 0.000 | 0.000 | 0.000 | 0.013 | 0.006 | 0.019 |
| SGT-L | Mean difference | 3.362^*^ | -1.026^*^ | 2.717^*^ | - | 0.396 | 0.792 | 1.434^*^ | 1.303^*^ | 1.514^*^ |
|  | Sig. | 0.000 | 0.045 | 0.000 | - | 0.436 | 0.120 | 0.006 | 0.011 | 0.003 |
| SGT-M | Mean difference | 2.966^*^ | -1.421^*^ | 2.322^*^ | -0.396 | - | 0.397 | 1.039^*^ | 0.907 | 1.118^*^ |
|  | Sig. | 0.000 | 0.006 | 0.000 | 0.436 | - | 0.434 | 0.043 | 0.076 | 0.029 |
| SGT-H | Mean difference | 2.569^*^ | -1.818^*^ | 1.925^*^ | -0.792 | -0.397 | - | 0.642 | 0.511 | 0.721 |
|  | Sig. | 0.000 | 0.001 | 0.000 | 0.120 | 0.434 | - | 0.207 | 0.315 | 0.157 |
| MPT-L | Mean difference | 1.927^*^ | -2.460^*^ | 1.283^*^ | -1.434^*^ | -1.039^*^ | -0.642 | - | -0.131 | 0.080 |
|  | Sig. | 0.000 | 0.000 | 0.013 | 0.006 | 0.043 | 0.207 | - | 0.795 | 0.875 |
| MPT-M | Mean difference | 2.059^*^ | -2.329^*^ | 1.414^*^ | -1.303^*^ | -0.907 | -0.511 | 0.131 | - | 0.211 |
|  | Sig. | 0.000 | 0.000 | 0.006 | 0.011 | 0.076 | 0.315 | 0.795 | - | 0.677 |
| MPT-H | Mean difference | 1.848^*^ | -2.540^*^ | 1.203^*^ | -1.514^*^ | -1.118^*^ | -0.721 | -0.080 | -0.211 | - |
|  | Sig. | 0.000 | 0.000 | 0.019 | 0.003 | 0.029 | 0.157 | 0.875 | 0.677 | - |

Notes: TC: total cholesterol, HFD: high-fat diet group, SGT-L: low dose of sun-dried green tea water extract group, SGT-M: medium dose of sun-dried green tea water extract group, SGT-H: high dose of sun-dried green tea water extract group, MPT-L: low dose of *Monascus purpureus* fermented pu-erh tea water extract group, MPT-M: medium dose of *Monascus purpureus* fermented pu-erh tea water extract group, MPT-H: high dose of *Monascus purpureus* fermented pu-erh tea water extract group.

Table S2 One-way ANOVA of serum TG in rats.

|  | | Control | HFD | Atorvastatin | SGT-L | SGT-M | SGT-H | MPT-L | MPT-M | MPT-H |
| --- | --- | --- | --- | --- | --- | --- | --- | --- | --- | --- |
| Control | Mean difference | - | -2.723^*^ | -0.358 | -2.077^*^ | -1.756^*^ | -1.103^*^ | -.925^*^ | -.681^*^ | -.597^*^ |
|  | Sig. | - | 0.000 | 0.157 | 0.000 | 0.000 | 0.000 | 0.000 | 0.008 | 0.019 |
| HFD | Mean difference | 2.723^*^ | - | 2.365^*^ | .647^*^ | .967^*^ | 1.620^*^ | 1.798^*^ | 2.042^*^ | 2.126^*^ |
|  | Sig. | 0.000 | - | 0.000 | 0.012 | 0.000 | 0.000 | 0.000 | 0.000 | 0.000 |
|  | Decreasing amplitude | -76.11% | - | -66.10% | -18.08% | -27.03% | -45.29% | -50.25% | -57.08% | -59.43% |
| Atorvastatin | Mean difference | 0.358 | -2.365^*^ | - | -1.718^*^ | -1.398^*^ | -.745^*^ | -.567^*^ | -0.323 | -0.239 |
|  | Sig. | 0.157 | 0.000 | - | 0.000 | 0.000 | 0.004 | 0.026 | 0.201 | 0.344 |
| SGT-L | Mean difference | 2.077^*^ | -.647^*^ | 1.718^*^ | - | 0.320 | .974^*^ | 1.151^*^ | 1.395^*^ | 1.480^*^ |
|  | Sig. | 0.000 | 0.012 | 0.000 | - | 0.205 | 0.000 | 0.000 | 0.000 | 0.000 |
| SGT-M | Mean difference | 1.756^*^ | -.967^*^ | 1.398^*^ | -0.320 | - | .653^*^ | .831^*^ | 1.075^*^ | 1.159^*^ |
|  | Sig. | 0.000 | 0.000 | 0.000 | 0.205 | - | 0.011 | 0.001 | 0.000 | 0.000 |
| SGT-H | Mean difference | 1.103^*^ | -1.620^*^ | .745^*^ | -.974^*^ | -.653^*^ | - | 0.177 | 0.422 | .506^*^ |
|  | Sig. | 0.000 | 0.000 | 0.004 | 0.000 | 0.011 | - | 0.481 | 0.096 | 0.047 |
| MPT-L | Mean difference | .925^*^ | -1.798^*^ | .567^*^ | -1.151^*^ | -.831^*^ | -0.177 | - | 0.244 | 0.329 |
|  | Sig. | 0.000 | 0.000 | 0.026 | 0.000 | 0.001 | 0.481 | - | 0.333 | 0.194 |
| MPT-M | Mean difference | .681^*^ | -2.042^*^ | 0.323 | -1.395^*^ | -1.075^*^ | -0.422 | -0.244 | - | 0.084 |
|  | Sig. | 0.008 | 0.000 | 0.201 | 0.000 | 0.000 | 0.096 | 0.333 | - | 0.737 |
| MPT-H | Mean difference | .597^*^ | -2.126^*^ | 0.239 | -1.480^*^ | -1.159^*^ | -.506^*^ | -0.329 | -0.084 | - |
|  | Sig. | 0.019 | 0.000 | 0.344 | 0.000 | 0.000 | 0.047 | 0.194 | 0.737 | - |

Notes: TG: Triacylglycerol, HFD: high-fat diet group, SGT-L: low dose of sun-dried green tea water extract group, SGT-M: medium dose of sun-dried green tea water extract group, SGT-H: high dose of sun-dried green tea water extract group, MPT-L: low dose of *Monascus purpureus* fermented pu-erh tea water extract group, MPT-M: medium dose of *Monascus purpureus* fermented pu-erh tea water extract group, MPT-H: high dose of *Monascus purpureus* fermented pu-erh tea water extract group.

Table S3 One-way ANOVA of serum LDL-C in rats.

|  | | Control | HFD | Atorvastatin | SGT-L | SGT-M | SGT-H | MPT-L | MPT-M | MPT-H |
| --- | --- | --- | --- | --- | --- | --- | --- | --- | --- | --- |
| Control | Mean difference | - | -2.808^*^ | -0.413 | -2.152^*^ | -1.851^*^ | -1.557^*^ | -1.234^*^ | -1.279^*^ | -1.110^*^ |
|  | Sig. | - | 0.000 | 0.198 | 0.000 | 0.000 | 0.000 | 0.000 | 0.000 | 0.001 |
| HFD | Mean difference | 2.808^*^ | - | 2.395^*^ | .656^*^ | .957^*^ | 1.251^*^ | 1.574^*^ | 1.529^*^ | 1.698^*^ |
|  | Sig. | 0.000 | - | 0.000 | 0.042 | 0.003 | 0.000 | 0.000 | 0.000 | 0.000 |
|  | Decreasing amplitude | -71.13% | - | -60.68% | -16.64% | -24.25% | -31.69% | -39.89% | -38.73% | -43.01% |
| Atorvastatin | Mean difference | 0.413 | -2.395^*^ | - | -1.739^*^ | -1.438^*^ | -1.145^*^ | -.821^*^ | -.867^*^ | -.698^*^ |
|  | Sig. | 0.198 | 0.000 | - | 0.000 | 0.000 | 0.001 | 0.011 | 0.008 | 0.031 |
| SGT-L | Mean difference | 2.152^*^ | -.656^*^ | 1.739^*^ | - | 0.301 | 0.594 | .918^*^ | .872^*^ | 1.041^*^ |
|  | Sig. | 0.000 | 0.042 | 0.000 | - | 0.347 | 0.065 | 0.005 | 0.007 | 0.001 |
| SGT-M | Mean difference | 1.851^*^ | -.957^*^ | 1.438^*^ | -0.301 | - | 0.294 | 0.617 | 0.572 | .741^*^ |
|  | Sig. | 0.000 | 0.003 | 0.000 | 0.347 | - | 0.359 | 0.055 | 0.076 | 0.022 |
| SGT-H | Mean difference | 1.557^*^ | -1.251^*^ | 1.145^*^ | -0.594 | -0.294 | - | 0.324 | 0.278 | 0.447 |
|  | Sig. | 0.000 | 0.000 | 0.001 | 0.065 | 0.359 | - | 0.312 | 0.385 | 0.163 |
| MPT-L | Mean difference | 1.234^*^ | -1.574^*^ | .821^*^ | -.918^*^ | -0.617 | -0.324 | - | -0.046 | 0.123 |
|  | Sig. | 0.000 | 0.000 | 0.011 | 0.005 | 0.055 | 0.312 | - | 0.886 | 0.699 |
| MPT-M | Mean difference | 1.279^*^ | -1.529^*^ | .867^*^ | -.872^*^ | -0.572 | -0.278 | 0.046 | - | 0.169 |
|  | Sig. | 0.000 | 0.000 | 0.008 | 0.007 | 0.076 | 0.385 | 0.886 | - | 0.597 |
| MPT-H | Mean difference | 1.110^*^ | -1.698^*^ | .698^*^ | -1.041^*^ | -.741^*^ | -0.447 | -0.123 | -0.169 | - |
|  | Sig. | 0.001 | 0.000 | 0.031 | 0.001 | 0.022 | 0.163 | 0.699 | 0.597 | - |

Notes: LDL-C: low-density lipoprotein cholesterol, HFD: high-fat diet group, SGT-L: low dose of sun-dried green tea water extract group, SGT-M: medium dose of sun-dried green tea water extract group, SGT-H: high dose of sun-dried green tea water extract group, MPT-L: low dose of *Monascus purpureus* fermented pu-erh tea water extract group, MPT-M: medium dose of *Monascus purpureus* fermented pu-erh tea water extract group, MPT-H: high dose of *Monascus purpureus* fermented pu-erh tea water extract group.

Table S4 One-way ANOVA of serum HDL-C in rats.

|  | | Control | HFD | Atorvastatin | SGT-L | SGT-M | SGT-H | MPT-L | MPT-M | MPT-H |
| --- | --- | --- | --- | --- | --- | --- | --- | --- | --- | --- |
| Control | Mean difference | - | 2.236^*^ | .888^*^ | 1.258^*^ | 1.352^*^ | 1.456^*^ | 1.774^*^ | 1.688^*^ | 1.730^*^ |
|  | Sig. | - | 0.000 | 0.000 | 0.000 | 0.000 | 0.000 | 0.000 | 0.000 | 0.000 |
| HFD | Mean difference | -2.236^*^ | - | -1.348^*^ | -.978^*^ | -.884^*^ | -.780^*^ | -.462^*^ | -.548^*^ | -.506^*^ |
|  | Sig. | 0.000 | - | 0.000 | 0.000 | 0.000 | 0.001 | 0.036 | 0.013 | 0.022 |
|  | Increasing amplitude | 256.11% | - | 154.37% | 112.07% | 101.20% | 89.37% | 52.91% | 62.73% | 57.94% |
| Atorvastatin | Mean difference | -.888^*^ | 1.348^*^ | - | 0.369 | .464^*^ | .568^*^ | .886^*^ | .800^*^ | .842^*^ |
|  | Sig. | 0.000 | 0.000 | - | 0.092 | 0.035 | 0.010 | 0.000 | 0.000 | 0.000 |
| SGT-L | Mean difference | -1.258^*^ | .978^*^ | -0.369 | - | 0.095 | 0.198 | .516^*^ | .431^*^ | .472^*^ |
|  | Sig. | 0.000 | 0.000 | 0.092 | - | 0.663 | 0.363 | 0.019 | 0.050 | 0.032 |
| SGT-M | Mean difference | -1.352^*^ | .884^*^ | -.464^*^ | -0.095 | - | 0.103 | 0.422 | 0.336 | 0.378 |
|  | Sig. | 0.000 | 0.000 | 0.035 | 0.663 | - | 0.635 | 0.055 | 0.125 | 0.085 |
| SGT-H | Mean difference | -1.456^*^ | .780^*^ | -.568^*^ | -0.198 | -0.103 | - | 0.318 | 0.232 | 0.274 |
|  | Sig. | 0.000 | 0.001 | 0.010 | 0.363 | 0.635 | - | 0.145 | 0.286 | 0.209 |
| MPT-L | Mean difference | -1.774^*^ | .462^*^ | -.886^*^ | -.516^*^ | -0.422 | -0.318 | - | -0.086 | -0.044 |
|  | Sig. | 0.000 | 0.036 | 0.000 | 0.019 | 0.055 | 0.145 | - | 0.693 | 0.840 |
| MPT-M | Mean difference | -1.688^*^ | .548^*^ | -.800^*^ | -.431^*^ | -0.336 | -0.232 | 0.086 | - | 0.042 |
|  | Sig. | 0.000 | 0.013 | 0.000 | 0.050 | 0.125 | 0.286 | 0.693 | - | 0.847 |
| MPT-H | Mean difference | -1.730^*^ | .506^*^ | -.842^*^ | -.472^*^ | -0.378 | -0.274 | 0.044 | -0.042 | - |
|  | Sig. | 0.000 | 0.022 | 0.000 | 0.032 | 0.085 | 0.209 | 0.840 | 0.847 | - |

Notes: HDL-C: high-density lipoprotein cholesterol, HFD: high-fat diet group, SGT-L: low dose of sun-dried green tea water extract group, SGT-M: medium dose of sun-dried green tea water extract group, SGT-H: high dose of sun-dried green tea water extract group, MPT-L: low dose of *Monascus purpureus* fermented pu-erh tea water extract group, MPT-M: medium dose of *Monascus purpureus* fermented pu-erh tea water extract group, MPT-H: high dose of *Monascus purpureus* fermented pu-erh tea water extract group.

Table S5 One-way ANOVA of liver TC in rats.

|  | | Control | HFD | Atorvastatin | SGT-L | SGT-M | SGT-H | MPT-L | MPT-M | MPT-H |
| --- | --- | --- | --- | --- | --- | --- | --- | --- | --- | --- |
| Control | Mean difference | - | -16.673^*^ | -2.449 | -9.586^*^ | -8.328^*^ | -7.066^*^ | -5.025^*^ | -5.443^*^ | -4.772^*^ |
|  | Sig. | - | 0.000 | 0.152 | 0.000 | 0.000 | 0.000 | 0.004 | 0.002 | 0.006 |
| HFD | Mean difference | 16.673^*^ | - | 14.224^*^ | 7.087^*^ | 8.344^*^ | 9.606^*^ | 11.648^*^ | 11.230^*^ | 11.901^*^ |
|  | Sig. | 0.000 | - | 0.000 | 0.000 | 0.000 | 0.000 | 0.000 | 0.000 | 0.000 |
|  | Decreasing amplitude | -71.13% | - | -60.68% | -30.23% | -35.60% | -40.98% | -49.69% | -47.91% | -50.77% |
| Atorvastatin | Mean difference | 2.449 | -14.224^*^ | - | -7.137^*^ | -5.880^*^ | -4.617^*^ | -2.576 | -2.994 | -2.323 |
|  | Sig. | 0.152 | 0.000 | - | 0.000 | 0.001 | 0.008 | 0.132 | 0.080 | 0.173 |
| SGT-L | Mean difference | 9.586^*^ | -7.087^*^ | 7.137^*^ | - | 1.258 | 2.520 | 4.561^*^ | 4.143^*^ | 4.814^*^ |
|  | Sig. | 0.000 | 0.000 | 0.000 | - | 0.460 | 0.140 | 0.008 | 0.016 | 0.005 |
| SGT-M | Mean difference | 8.328^*^ | -8.344^*^ | 5.880^*^ | -1.258 | - | 1.262 | 3.303 | 2.886 | 3.557^*^ |
|  | Sig. | 0.000 | 0.000 | 0.001 | 0.460 | - | 0.458 | 0.054 | 0.092 | 0.038 |
| SGT-H | Mean difference | 7.066^*^ | -9.606^*^ | 4.617^*^ | -2.520 | -1.262 | - | 2.041 | 1.623 | 2.294 |
|  | Sig. | 0.000 | 0.000 | 0.008 | 0.140 | 0.458 | - | 0.231 | 0.340 | 0.179 |
| MPT-L | Mean difference | 5.025^*^ | -11.648^*^ | 2.576 | -4.561^*^ | -3.303 | -2.041 | - | -0.418 | 0.253 |
|  | Sig. | 0.004 | 0.000 | 0.132 | 0.008 | 0.054 | 0.231 | - | 0.806 | 0.881 |
| MPT-M | Mean difference | 5.443^*^ | -11.230^*^ | 2.994 | -4.143^*^ | -2.886 | -1.623 | 0.418 | - | 0.671 |
|  | Sig. | 0.002 | 0.000 | 0.080 | 0.016 | 0.092 | 0.340 | 0.806 | - | 0.693 |
| MPT-H | Mean difference | 4.772^*^ | -11.901^*^ | 2.323 | -4.814^*^ | -3.557^*^ | -2.294 | -0.253 | -0.671 | - |
|  | Sig. | 0.006 | 0.000 | 0.173 | 0.005 | 0.038 | 0.179 | 0.881 | 0.693 | - |

Notes: TC: total cholesterol, HFD: high-fat diet group, SGT-L: low dose of sun-dried green tea water extract group, SGT-M: medium dose of sun-dried green tea water extract group, SGT-H: high dose of sun-dried green tea water extract group, MPT-L: low dose of *Monascus purpureus* fermented pu-erh tea water extract group, MPT-M: medium dose of *Monascus purpureus* fermented pu-erh tea water extract group, MPT-H: high dose of *Monascus purpureus* fermented pu-erh tea water extract group.

Table S6 One-way ANOVA of liver TG in rats.

|  | | Control | HFD | Atorvastatin | SGT-L | SGT-M | SGT-H | MPT-L | MPT-M | MPT-H |
| --- | --- | --- | --- | --- | --- | --- | --- | --- | --- | --- |
| Control | Mean difference | - | -30.229^*^ | -5.341 | -19.696^*^ | -16.717^*^ | -18.650^*^ | -14.907^*^ | -10.920^*^ | -9.509^*^ |
|  | Sig. | - | 0.000 | 0.178 | 0.000 | 0.000 | 0.000 | 0.000 | 0.007 | 0.018 |
| HFD | Mean difference | 30.229^*^ | - | 24.888^*^ | 10.534^*^ | 13.512^*^ | 11.580^*^ | 15.323^*^ | 19.309^*^ | 20.720^*^ |
|  | Sig. | 0.000 | - | 0.000 | 0.009 | 0.001 | 0.004 | 0.000 | 0.000 | 0.000 |
|  | Decreasing amplitude | -67.19% | - | -55.32% | -23.41% | -30.03% | -25.74% | -34.06% | -42.92% | -46.06% |
| Atorvastatin | Mean difference | 5.341 | -24.888^*^ | - | -14.354^*^ | -11.376^*^ | -13.308^*^ | -9.565^*^ | -5.579 | -4.168 |
|  | Sig. | 0.178 | 0.000 | - | 0.000 | 0.005 | 0.001 | 0.017 | 0.160 | 0.293 |
| SGT-L | Mean difference | 19.696^*^ | -10.534^*^ | 14.354^*^ | - | 2.979 | 1.046 | 4.789 | 8.776^*^ | 10.187^*^ |
|  | Sig. | 0.000 | 0.009 | 0.000 | - | 0.451 | 0.791 | 0.227 | 0.028 | 0.011 |
| SGT-M | Mean difference | 16.717^*^ | -13.512^*^ | 11.376^*^ | -2.979 | - | -1.933 | 1.811 | 5.797 | 7.208 |
|  | Sig. | 0.000 | 0.001 | 0.005 | 0.451 | - | 0.625 | 0.647 | 0.144 | 0.070 |
| SGT-H | Mean difference | 18.650^*^ | -11.580^*^ | 13.308^*^ | -1.046 | 1.933 | - | 3.743 | 7.730 | 9.141^*^ |
|  | Sig. | 0.000 | 0.004 | 0.001 | 0.791 | 0.625 | - | 0.344 | 0.052 | 0.022 |
| MPT-L | Mean difference | 14.907^*^ | -15.323^*^ | 9.565^*^ | -4.789 | -1.811 | -3.743 | - | 3.986 | 5.397 |
|  | Sig. | 0.000 | 0.000 | 0.017 | 0.227 | 0.647 | 0.344 | - | 0.314 | 0.174 |
| MPT-M | Mean difference | 10.920^*^ | -19.309^*^ | 5.579 | -8.776^*^ | -5.797 | -7.730 | -3.986 | - | 1.411 |
|  | Sig. | 0.007 | 0.000 | 0.160 | 0.028 | 0.144 | 0.052 | 0.314 | - | 0.721 |
| MPT-H | Mean difference | 9.509^*^ | -20.720^*^ | 4.168 | -10.187^*^ | -7.208 | -9.141^*^ | -5.397 | -1.411 | - |
|  | Sig. | 0.018 | 0.000 | 0.293 | 0.011 | 0.070 | 0.022 | 0.174 | 0.721 | - |

Notes: TG: Triacylglycerol, HFD: high-fat diet group, SGT-L: low dose of sun-dried green tea water extract group, SGT-M: medium dose of sun-dried green tea water extract group, SGT-H: high dose of sun-dried green tea water extract group, MPT-L: low dose of *Monascus purpureus* fermented pu-erh tea water extract group, MPT-M: medium dose of *Monascus purpureus* fermented pu-erh tea water extract group, MPT-H: high dose of *Monascus purpureus* fermented pu-erh tea water extract group.

Table S7 One-way ANOVA of serum inflammatory cytokines IL-1β in rats.

|  | | Control | HFD | Atorvastatin | SGT-L | SGT-M | SGT-H | MPT-L | MPT-M | MPT-H |
| --- | --- | --- | --- | --- | --- | --- | --- | --- | --- | --- |
| Control | Mean difference | - | -47.773^*^ | -31.528^*^ | -40.059^*^ | -18.128^*^ | -12.733 | -18.598^*^ | -23.321^*^ | -13.069 |
|  | Sig. | - | 0.000 | 0.000 | 0.000 | 0.012 | 0.075 | 0.010 | 0.001 | 0.067 |
| HFD | Mean difference | 47.773^*^ | - | 16.244^*^ | 7.713 | 29.645^*^ | 35.039^*^ | 29.174^*^ | 24.452^*^ | 34.703^*^ |
|  | Sig. | 0.000 | - | 0.024 | 0.278 | 0.000 | 0.000 | 0.000 | 0.001 | 0.000 |
|  | Decreasing amplitude | -51.37% | - | -17.47% | -8.29% | -31.87% | -37.67% | -31.37% | -26.29% | -37.31% |
| Atorvastatin | Mean difference | 31.528^*^ | -16.244^*^ | - | -8.531 | 13.400 | 18.795^*^ | 12.930 | 8.207 | 18.459^*^ |
|  | Sig. | 0.000 | 0.024 | - | 0.230 | 0.061 | 0.009 | 0.070 | 0.248 | 0.010 |
| SGT-L | Mean difference | 40.059^*^ | -7.713 | 8.531 | - | 21.931^*^ | 27.326^*^ | 21.461^*^ | 16.738^*^ | 26.990^*^ |
|  | Sig. | 0.000 | 0.278 | 0.230 | - | 0.002 | 0.000 | 0.003 | 0.020 | 0.000 |
| SGT-M | Mean difference | 18.128^*^ | -29.645^*^ | -13.400 | -21.931^*^ | - | 5.395 | -0.471 | -5.193 | 5.058 |
|  | Sig. | 0.012 | 0.000 | 0.061 | 0.002 | - | 0.447 | 0.947 | 0.464 | 0.476 |
| SGT-H | Mean difference | 12.733 | -35.039^*^ | -18.795^*^ | -27.326^*^ | -5.395 | - | -5.865 | -10.588 | -0.336 |
|  | Sig. | 0.075 | 0.000 | 0.009 | 0.000 | 0.447 | - | 0.409 | 0.137 | 0.962 |
| MPT-L | Mean difference | 18.598^*^ | -29.174^*^ | -12.930 | -21.461^*^ | 0.471 | 5.865 | - | -4.722 | 5.529 |
|  | Sig. | 0.010 | 0.000 | 0.070 | 0.003 | 0.947 | 0.409 | - | 0.505 | 0.436 |
| MPT-M | Mean difference | 23.321^*^ | -24.452^*^ | -8.207 | -16.738^*^ | 5.193 | 10.588 | 4.722 | - | 10.251 |
|  | Sig. | 0.001 | 0.001 | 0.248 | 0.020 | 0.464 | 0.137 | 0.505 | - | 0.150 |
| MPT-H | Mean difference | 13.069 | -34.703^*^ | -18.459^*^ | -26.990^*^ | -5.058 | 0.336 | -5.529 | -10.251 | - |
|  | Sig. | 0.067 | 0.000 | 0.010 | 0.000 | 0.476 | 0.962 | 0.436 | 0.150 | - |

Notes: HFD: high-fat diet group, SGT-L: low dose of sun-dried green tea water extract group, SGT-M: medium dose of sun-dried green tea water extract group, SGT-H: high dose of sun-dried green tea water extract group, MPT-L: low dose of *Monascus purpureus* fermented pu-erh tea water extract group, MPT-M: medium dose of *Monascus purpureus* fermented pu-erh tea water extract group, MPT-H: high dose of *Monascus purpureus* fermented pu-erh tea water extract group.

Table S8 One-way ANOVA of serum inflammatory cytokines IL-6 in rats.

|  | | Control | HFD | Atorvastatin | SGT-L | SGT-M | SGT-H | MPT-L | MPT-M | MPT-H |
| --- | --- | --- | --- | --- | --- | --- | --- | --- | --- | --- |
| Control | Mean difference | - | -516.896^*^ | -173.057^*^ | -320.217^*^ | -163.566^*^ | -116.914 | -109.890 | -129.350 | 21.394 |
|  | Sig. | - | 0.000 | 0.034 | 0.000 | 0.045 | 0.150 | 0.175 | 0.111 | 0.791 |
| HFD | Mean difference | 516.896^*^ | - | 343.839^*^ | 196.679^*^ | 353.330^*^ | 399.982^*^ | 407.006^*^ | 387.546^*^ | 538.290^*^ |
|  | Sig. | 0.000 | - | 0.000 | 0.016 | 0.000 | 0.000 | 0.000 | 0.000 | 0.000 |
|  | Decreasing amplitude | -51.89% | - | -34.52% | -19.75% | -35.47% | -40.16% | -40.86% | -38.91% | -54.04% |
| Atorvastatin | Mean difference | 173.057^*^ | -343.839^*^ | - | -147.161 | 9.491 | 56.143 | 63.167 | 43.707 | 194.451^*^ |
|  | Sig. | 0.034 | 0.000 | - | 0.071 | 0.906 | 0.487 | 0.434 | 0.588 | 0.018 |
| SGT-L | Mean difference | 320.217^*^ | -196.679^*^ | 147.161 | - | 156.651 | 203.303^*^ | 210.327^*^ | 190.868^*^ | 341.612^*^ |
|  | Sig. | 0.000 | 0.016 | 0.071 | - | 0.054 | 0.013 | 0.010 | 0.020 | 0.000 |
| SGT-M | Mean difference | 163.566^*^ | -353.330^*^ | -9.491 | -156.651 | - | 46.652 | 53.676 | 34.216 | 184.960^*^ |
|  | Sig. | 0.045 | 0.000 | 0.906 | 0.054 | - | 0.564 | 0.506 | 0.672 | 0.024 |
| SGT-H | Mean difference | 116.914 | -399.982^*^ | -56.143 | -203.303^*^ | -46.652 | - | 7.024 | -12.436 | 138.308 |
|  | Sig. | 0.150 | 0.000 | 0.487 | 0.013 | 0.564 | - | 0.931 | 0.878 | 0.089 |
| MPT-L | Mean difference | 109.890 | -407.006^*^ | -63.167 | -210.327^*^ | -53.676 | -7.024 | - | -19.460 | 131.284 |
|  | Sig. | 0.175 | 0.000 | 0.434 | 0.010 | 0.506 | 0.931 | - | 0.809 | 0.106 |
| MPT-M | Mean difference | 129.350 | -387.546^*^ | -43.707 | -190.868^*^ | -34.216 | 12.436 | 19.460 | - | 150.744 |
|  | Sig. | 0.111 | 0.000 | 0.588 | 0.020 | 0.672 | 0.878 | 0.809 | - | 0.064 |
| MPT-H | Mean difference | -21.394 | -538.290^*^ | -194.451^*^ | -341.612^*^ | -184.960^*^ | -138.308 | -131.284 | -150.744 | - |
|  | Sig. | 0.791 | 0.000 | 0.018 | 0.000 | 0.024 | 0.089 | 0.106 | 0.064 | - |

Notes: HFD: high-fat diet group, SGT-L: low dose of sun-dried green tea water extract group, SGT-M: medium dose of sun-dried green tea water extract group, SGT-H: high dose of sun-dried green tea water extract group, MPT-L: low dose of *Monascus purpureus* fermented pu-erh tea water extract group, MPT-M: medium dose of *Monascus purpureus* fermented pu-erh tea water extract group, MPT-H: high dose of *Monascus purpureus* fermented pu-erh tea water extract group.

Table S9 One-way ANOVA of serum inflammatory cytokines TNF-α in rats.

|  | | Control | HFD | Atorvastatin | SGT-L | SGT-M | SGT-H | MPT-L | MPT-M | MPT-H |
| --- | --- | --- | --- | --- | --- | --- | --- | --- | --- | --- |
| Control | Mean difference | - | -526.034^*^ | -251.802^*^ | -504.957^*^ | -293.188^*^ | -274.491^*^ | -266.529^*^ | -288.588^*^ | -117.704 |
|  | Sig. | - | 0.000 | 0.004 | 0.000 | 0.001 | 0.002 | 0.002 | 0.001 | 0.172 |
| HFD | Mean difference | 526.034^*^ | - | 274.232^*^ | 21.077 | 232.845^*^ | 251.542^*^ | 259.505^*^ | 237.445^*^ | 408.329^*^ |
|  | Sig. | 0.000 | - | 0.002 | 0.806 | 0.008 | 0.004 | 0.003 | 0.007 | 0.000 |
|  | Decreasing amplitude | -50.44% | - | -26.30% | -2.02% | -22.33% | -24.12% | -24.88% | -22.77% | -39.15% |
| Atorvastatin | Mean difference | 251.802^*^ | -274.232^*^ | - | -253.155^*^ | -41.386 | -22.689 | -14.727 | -36.786 | 134.098 |
|  | Sig. | 0.004 | 0.002 | - | 0.004 | 0.630 | 0.791 | 0.864 | 0.668 | 0.120 |
| SGT-L | Mean difference | 504.957^*^ | -21.077 | 253.155^*^ | - | 211.768^*^ | 230.465^*^ | 238.428^*^ | 216.368^*^ | 387.252^*^ |
|  | Sig. | 0.000 | 0.806 | 0.004 | - | 0.015 | 0.008 | 0.006 | 0.013 | 0.000 |
| SGT-M | Mean difference | 293.188^*^ | -232.845^*^ | 41.386 | -211.768^*^ | - | 18.697 | 26.659 | 4.600 | 175.484^*^ |
|  | Sig. | 0.001 | 0.008 | 0.630 | 0.015 | - | 0.827 | 0.756 | 0.957 | 0.043 |
| SGT-H | Mean difference | 274.491^*^ | -251.542^*^ | 22.689 | -230.465^*^ | -18.697 | - | 7.963 | -14.097 | 156.787 |
|  | Sig. | 0.002 | 0.004 | 0.791 | 0.008 | 0.827 | - | 0.926 | 0.869 | 0.070 |
| MPT-L | Mean difference | 266.529^*^ | -259.505^*^ | 14.727 | -238.428^*^ | -26.659 | -7.963 | - | -22.060 | 148.824 |
|  | Sig. | 0.002 | 0.003 | 0.864 | 0.006 | 0.756 | 0.926 | - | 0.797 | 0.085 |
| MPT-M | Mean difference | 288.588^*^ | -237.445^*^ | 36.786 | -216.368^*^ | -4.600 | 14.097 | 22.060 | - | 170.884^*^ |
|  | Sig. | 0.001 | 0.007 | 0.668 | 0.013 | 0.957 | 0.869 | 0.797 | - | 0.049 |
| MPT-H | Mean difference | 117.704 | -408.329^*^ | -134.098 | -387.252^*^ | -175.484^*^ | -156.787 | -148.824 | -170.884^*^ | - |
|  | Sig. | 0.172 | 0.000 | 0.120 | 0.000 | 0.043 | 0.070 | 0.085 | 0.049 | - |

Notes: HFD: high-fat diet group, SGT-L: low dose of sun-dried green tea water extract group, SGT-M: medium dose of sun-dried green tea water extract group, SGT-H: high dose of sun-dried green tea water extract group, MPT-L: low dose of *Monascus purpureus* fermented pu-erh tea water extract group, MPT-M: medium dose of *Monascus purpureus* fermented pu-erh tea water extract group, MPT-H: high dose of *Monascus purpureus* fermented pu-erh tea water extract group.

Table S10 One-way ANOVA of human micro-artery endothelial cells(HMEC) survival number.

|  | | Control | LPS | Atorvastatin | SGT-L | SGT-M | SGT-H | MPT-L | MPT-M | MPT-H |
| --- | --- | --- | --- | --- | --- | --- | --- | --- | --- | --- |
| Control | Mean difference | - | 0.210^*^ | 0.155^*^ | 0.205^*^ | 0.153^*^ | 0.061^*^ | 0.177^*^ | 0.107^*^ | 0.044^*^ |
|  | Sig. | - | 0.000 | 0.000 | 0.000 | 0.000 | 0.000 | 0.000 | 0.000 | 0.003 |
| LPS | Mean difference | -0.210^*^ | - | -0.055^*^ | -0.006 | -0.057^*^ | -0.149^*^ | -0.034^*^ | -0.103^*^ | -0.166^*^ |
|  | Sig. | 0.000 | - | 0.000 | 0.697 | 0.000 | 0.000 | 0.021 | 0.000 | 0.000 |
|  | Decreasing amplitude | 104.49% | - | 27.55% | 2.75% | 28.56% | 74.30% | 16.66% | 51.20% | 82.59% |
| Atorvastatin | Mean difference | -0.155^*^ | 0.055^*^ | - | 0.050^*^ | -0.002 | -0.094^*^ | 0.022 | -0.048^*^ | -0.111^*^ |
|  | Sig. | 0.000 | 0.000 | - | 0.001 | 0.886 | 0.000 | 0.127 | 0.001 | 0.000 |
| SGT-L | Mean difference | -0.205^*^ | 0.006 | -0.050^*^ | - | -0.052^*^ | -0.144^*^ | -0.028 | -0.097^*^ | -0.161^*^ |
|  | Sig. | 0.000 | 0.697 | 0.001 | - | 0.001 | 0.000 | 0.053 | 0.000 | 0.000 |
| SGT-M | Mean difference | -0.153^*^ | 0.057^*^ | 0.002 | 0.052^*^ | - | -0.092^*^ | 0.024 | -0.046^*^ | -0.109^*^ |
|  | Sig. | 0.000 | 0.000 | 0.886 | 0.001 | - | 0.000 | 0.096 | 0.002 | 0.000 |
| SGT-H | Mean difference | -0.061^*^ | 0.149^*^ | 0.094^*^ | 0.144^*^ | 0.092^*^ | - | 0.116^*^ | 0.046^*^ | -0.017 |
|  | Sig. | 0.000 | 0.000 | 0.000 | 0.000 | 0.000 | - | 0.000 | 0.002 | 0.244 |
| MPT-L | Mean difference | -0.177^*^ | 0.034^*^ | -0.022 | 0.028 | -0.024 | -0.116^*^ | - | -0.069^*^ | -0.133^*^ |
|  | Sig. | 0.000 | 0.021 | 0.127 | 0.053 | 0.096 | 0.000 | - | 0.000 | 0.000 |
| MPT-M | Mean difference | -0.107^*^ | 0.103^*^ | 0.048^*^ | 0.097^*^ | 0.046^*^ | -0.046^*^ | 0.069^*^ | - | -0.063^*^ |
|  | Sig. | 0.000 | 0.000 | 0.001 | 0.000 | 0.002 | 0.002 | 0.000 | - | 0.000 |
| MPT-H | Mean difference | -0.044^*^ | 0.166^*^ | 0.111^*^ | 0.161^*^ | 0.109^*^ | 0.017^*^ | 0.133^*^ | 0.063^*^ | - |
|  | Sig. | 0.003 | 0.000 | 0.000 | 0.000 | 0.000 | 0.244 | 0.000 | 0.000 | - |

Notes: LPS: [lipopolysaccharide](../../../../D:/Program%20Files%20(x86)/Youdao/Dict/8.6.2.0/resultui/html/index.html#/javascript:;) group, SGT-L: low dose of sun-dried green tea water extract group, SGT-M: medium dose of sun-dried green tea water extract group, SGT-H: high dose of sun-dried green tea water extract group, MPT-L: low dose of *Monascus purpureus* fermented pu-erh tea water extract group, MPT-M: medium dose of *Monascus purpureus* fermented pu-erh tea water extract group, MPT-H: high dose of *Monascus purpureus* fermented pu-erh tea water extract group.

Table S11 One-way ANOVA of arterial [thrombosis](../../../../D:/Program%20Files%20(x86)/Youdao/Dict/8.6.2.0/resultui/html/index.html#/javascript:;) time in rats.

|  | | Control | HFD | Atorvastatin | SGT-L | SGT-M | SGT-H | MPT-L | MPT-M | MPT-H |
| --- | --- | --- | --- | --- | --- | --- | --- | --- | --- | --- |
| Control | Mean difference | - | 8.690^*^ | 6.652^*^ | 7.355^*^ | 6.222^*^ | 2.512^*^ | 7.800^*^ | 4.127^*^ | 1.615 |
|  | Sig. | - | 0.000 | 0.000 | 0.000 | 0.000 | 0.033 | 0.000 | 0.001 | 0.167 |
| HFD | Mean difference | -8.690^*^ | - | -2.038 | -1.335 | -2.468^*^ | -6.178^*^ | -0.890 | -4.563^*^ | -7.075^*^ |
|  | Sig. | 0.000 | - | 0.082 | 0.252 | 0.036 | 0.000 | 0.444 | 0.000 | 0.000 |
|  | Decreasing amplitude | 74.51% | - | 17.48% | 11.45% | 21.16% | 52.97% | 7.63% | 39.13% | 60.66% |
| Atorvastatin | Mean difference | -6.652^*^ | 2.038 | - | 0.703 | -0.430 | -4.140^*^ | 1.148 | -2.525^*^ | -5.037^*^ |
|  | Sig. | 0.000 | 0.082 | - | 0.545 | 0.711 | 0.001 | 0.324 | 0.032 | 0.000 |
| SGT-L | Mean difference | -7.355^*^ | 1.335 | -0.703 | - | -1.133 | -4.843^*^ | 0.445 | -3.228^*^ | -5.740^*^ |
|  | Sig. | 0.000 | 0.252 | 0.545 | - | 0.331 | 0.000 | 0.702 | 0.007 | 0.000 |
| SGT-M | Mean difference | -6.222^*^ | 2.468^*^ | 0.430 | 1.133 | - | -3.710^*^ | 1.578 | -2.095 | -4.607^*^ |
|  | Sig. | 0.000 | 0.036 | 0.711 | 0.331 | - | 0.002 | 0.177 | 0.074 | 0.000 |
| SGT-H | Mean difference | -2.512^*^ | 6.178^*^ | 4.140^*^ | 4.843^*^ | 3.710^*^ | - | 5.288^*^ | 1.615 | -0.897 |
|  | Sig. | 0.033 | 0.000 | 0.001 | 0.000 | 0.002 | - | 0.000 | 0.167 | 0.441 |
| MPT-L | Mean difference | -7.800^*^ | 0.890 | -1.148 | -0.445 | -1.578 | 5.288^*^ | - | -3.673^*^ | -6.185^*^ |
|  | Sig. | 0.000 | 0.444 | 0.324 | 0.702 | 0.177 | 0.000 | - | 0.002 | 0.000 |
| MPT-M | Mean difference | -4.127^*^ | 4.563^*^ | 2.525^*^ | 3.228^*^ | 2.095 | -1.615 | 3.673^*^ | - | -2.512^*^ |
|  | Sig. | 0.001 | 0.000 | 0.032 | 0.007 | 0.074 | 0.167 | 0.002 | - | 0.033 |
| MPT-H | Mean difference | -1.615 | 7.075^*^ | 5.037^*^ | 5.740^*^ | 4.607^*^ | 0.897 | 6.185^*^ | 2.512^*^ | - |
|  | Sig. | 0.167 | 0.000 | 0.000 | 0.000 | 0.000 | 0.441 | 0.000 | 0.033 | - |

Notes: HFD: high-fat diet group, SGT-L: low dose of sun-dried green tea water extract group, SGT-M: medium dose of sun-dried green tea water extract group, SGT-H: high dose of sun-dried green tea water extract group, MPT-L: low dose of *Monascus purpureus* fermented pu-erh tea water extract group, MPT-M: medium dose of *Monascus purpureus* fermented pu-erh tea water extract group, MPT-H: high dose of *Monascus purpureus* fermented pu-erh tea water extract group.

Table S12 Correlation analysis of chemical composition and efficacy indicators.

|  | | [T](../../../../D:/Program%20Files%20(x86)/Youdao/Dict/8.9.3.0/resultui/html/index.html#/javascript:;)ea  [polyphenol](../../../../D:/Program%20Files%20(x86)/Youdao/Dict/8.9.3.0/resultui/html/index.html#/javascript:;) | Amino  acids | Flavonoids | Theabrownins | EGCG | Gallic acid | Lovastatin | Theaflavins | Thearubigins | Soluble  sugar | Caffeine | Catechins | EGC | EC | ECG |
| --- | --- | --- | --- | --- | --- | --- | --- | --- | --- | --- | --- | --- | --- | --- | --- | --- |
| IL-1β | Pearson correlation | -.492 | -.474 | -.634 | -.462 | -.242 | -.525 | -.362 | -.499 | -.466 | -.553 | -.706 | -.362 | -.410 | -.374 | -.242 |
|  | Sig. | .322 | .343 | .177 | .357 | .644 | .285 | .481 | .313 | .351 | .255 | .117 | .480 | .419 | .465 | .644 |
| IL-6 | Pearson correlation | -.173 | -.144 | -.854^*^ | -.819^*^ | .182 | -.844^*^ | -.762 | -.186 | -.133 | -.279 | -.669 | .022 | -.047 | .006 | .182 |
|  | Sig. | .743 | .785 | .030 | .046 | .731 | .035 | .078 | .725 | .802 | .593 | .147 | .967 | .929 | .992 | .731 |
| TNF-α | Pearson correlation | -.189 | -.161 | -.829^*^ | -.786 | .154 | -.813^*^ | -.728 | -.201 | -.150 | -.291 | -.661 | -.001 | -.068 | -.017 | .154 |
|  | Sig. | .719 | .760 | .041 | .064 | .771 | .049 | .101 | .702 | .776 | .576 | .153 | .998 | .899 | .975 | .771 |
| Survival  HMEC | Pearson correlation | .594 | .567 | .912^*^ | .710 | .231 | .786 | .584 | .606 | .557 | .688 | .951^**^ | .404 | .474 | .420 | .231 |
|  | Sig. | .213 | .240 | .011 | .114 | .660 | .064 | .223 | .202 | .251 | .131 | .004 | .427 | .343 | .407 | .660 |
| [T](../../../../D:/Program%20Files%20(x86)/Youdao/Dict/8.6.2.0/resultui/html/index.html#/javascript:;)hrombosis time | Pearson correlation | .592 | .564 | .909^*^ | .708 | .229 | .785 | .583 | .603 | .554 | .685 | .947^**^ | .401 | .471 | .418 | .229 |
|  | Sig. | .216 | .243 | .012 | .115 | .663 | .064 | .224 | .205 | .254 | .133 | .004 | .430 | .346 | .410 | .663 |

Notes: EC: (-)-epicatechin; EGC: (-)-epigallocatechin; ECG: (-)-epicatechin gallate; EGCG: (-)-epigallocatechin gallate. *. At the level of 0.05 (double tails), the correlation was significant. **. At 0.01 level (double-tailed), the correlation was significant.
